# ROMO1 as a Diagnostic Biomarker in Cervical Neoplasia: Evidence from Normal, Pre-Invasive, and Invasive Lesions

**DOI:** 10.3390/diagnostics16010024

**Published:** 2025-12-21

**Authors:** Eva Tsoneva, Polina Damyanova, Metodi V. Metodiev, Velizar Shivarov, Mariela Vasileva-Slaveva, Zornitsa Gorcheva, Yonka Ivanova, Yavor Kornovski, Stoyan Kostov, Stanislav Slavchev, Margarita Nikolova, Angel Yordanov, Rafał Watrowski

**Affiliations:** 1 Department of Reproductive Medicine, Specialized Hospital for Active Treatment of Obstetrics and Gynaecology “Dr. Shterev”, 1330 Sofia, Bulgaria; 2 Faculty of Medicine, Medical University Pleven, 5800 Pleven, Bulgaria; 3 Department of General and Clinical Pathology, Heart and Brain Center of Clinical Excellence, 5800 Pleven, Bulgaria; poldamdim@abv.bg; 4 Life Sciences Laboratory, School of Biological Sciences, University of Essex, Wivenhoe Park, Colchester CO4 3SQ, UK; mmetod@essex.ac.uk; 5 Research Institute, Medical University Pleven, 5800 Pleven, Bulgaria; vshivarov@abv.bg (V.S.); drstoqn.kostov@gmail.com (S.K.); 6 Bulgarian Breast and Other Gynecological Cancers Association, 1750 Sofia, Bulgaria; sscvasileva@gmail.com; 7 Department of Breast Surgery, “Dr. Shterev” Hospital, 1750 Sofia, Bulgaria; 8 Department of Nephrology, Haematology and Gastroenterology, Medical University Pleven, 5800 Pleven, Bulgaria; zornica.gorchev@gmail.com; 9 Department of Gynecology, Hospital “Saint Anna”, Medical University—“Prof. Dr. Paraskev Stoyanov”, 9002 Varna, Bulgaria; yonka.ivanova@abv.bg (Y.I.); ykornovski@abv.bg (Y.K.); st_slavchev@abv.bg (S.S.); 10 Department of Pathology Laboratory, Saint Marina University Hospital, 5800 Pleven, Bulgaria; mnikol@abv.bg; 11 Department of Gynaecological Oncology, Medical University Pleven, 5800 Pleven, Bulgaria; 12 Department of Obstetrics and Gynecology, Helios Hospital Müllheim, 79379 Müllheim, Germany; rafal.watrowski@medizin.uni-freiburg.de; 13 Faculty of Medicine, University of Freiburg, 79106 Freiburg, Germany

**Keywords:** ROMO1, cervical carcinoma, cervical neoplasia, HPV

## Abstract

**Background**: Cervical cancer (CC) is the fourth most common malignancy in women around the world, with more than 600,000 new cases registered in 2022 and around 350,000 deaths. It is a growing social problem, especially in developing countries. Almost all cases of cervical cancer are caused by persistent infection with oncogenic high-risk human papillomavirus (HPV). This malignancy usually exhibits a gradual development through well-defined precursor stages, known as cervical intraepithelial neoplasia (CIN) grades 1, 2, and 3, before evolving into invasive carcinoma. In diagnostic practice, several biomarkers have been implemented to improve the detection of high-risk cervical lesions. p16 and Ki-67 greatly aid in identifying HPV-driven dysplasia, but they cannot always reliably distinguish progressive lesions from regressive or transient HPV infections. These limitations highlight the need for novel biomarkers with better predictive accuracy to complement current screening and diagnostic algorithms. ROMO1 has become a possible marker of a high-ROS, high-risk tumor phenotype in a number of cancers. Although oxidative stress, HPV, and cervical carcinogenesis have been linked, nothing is known about ROMO1’s involvement in cervical neoplasia. There is currently a lack of thorough information regarding the expression of ROMO1 in normal vs. precancerous lesions and in cervical cancer, as well as on whether or not its expression is correlated with the severity of the disease. In order to define ROMO1 expression throughout the course of cervical squamous neoplastic development, the current study was created. **Methods**: We performed immunohistochemical analysis of ROMO1 expression on cervical tissue samples from three groups: healthy cervix (*n* = 30), cervical intraepithelial neoplasia (CIN) (*n* = 41), and invasive cervical carcinoma (*n* = 205). ROMO1 expression in invasive carcinoma was evaluated using an H-score scale. **Results**: ROMO1 expression was basal in all normal cervix samples (0/30 cases). In contrast, CIN lesions showed 100% ROMO1 expression in the suprabasal layers of abnormal cells in all CIN cases. In invasive cervical carcinomas, ROMO1 expression was heterogeneous. In our cancer cohort (*n* = 205), ROMO1 H-score showed no significant association with the following: FIGO stage I vs. II vs. III (*p* = 0.25); histologic grade G1 vs. G2 vs. G3 (*p* = 0.46); lymphovascular invasion (no vs. yes; *p* = 0.80); nodal status N0 vs. N1 (*p* = 0.67); patient age (≤50 y vs. >50 y; *p* = 0.38). However, ROMO1 expression did vary by histologic subtype (AC vs. ASC vs. SCC; *p* = 0.02), with SCC enriched for strong staining compared to AC/ASC. With regard to tumor stage (pT stage), pT2a tumors exhibited significantly lower ROMO1 (pT1b1–pT2b; *p* = 0.035) than pT1b1 (*p* = 0.04). No other clinicopathologic variable remained significant. Notably, ROMO1 expression was highest in stage I tumors and declined in more advanced stages of cervical carcinoma. **Conclusions:** These results show a clear pattern of ROMO1 expression across the cervical neoplasia spectrum: it is attenuated in invasive tumors (with a peak in early-stage illness), significantly raised in pre-cancerous CIN lesions, and negligible in normal epithelium. The idea that oxidative stress may be the primary cause of early malignant transformation in the cervix is supported by the noticeable overexpression of ROMO1 in early lesions. For the detection of early-stage cervical carcinoma and high-grade precancerous lesions, ROMO1 may be a useful auxiliary biomarker.

## 1. Introduction

Cervical cancer (CC) is the fourth most common malignancy in women around the world, with more than 600,000 new cases registered in 2022 and around 350,000 deaths [1]. It is a growing social problem, especially in developing countries. According to the World Health Organization, the highest incidence and mortality rates of CC are in sub-Saharan Africa, Central America and Southeast Asia [1]. An estimated 210,000 children worldwide have lost their mothers to cervical cancer, representing roughly one-fifth of all cancer-related maternal orphanhood. The vast majority of these families reside in low- and middle-income regions, where cervical cancer mortality remains disproportionately high [2]. In Bulgaria, for example, there were 877 newly diagnosed cases and 453 deaths from CC in 2022 [3]. These statistics highlight the urgent need for improved prevention and diagnostic strategies.

Almost all cases of cervical cancer are caused by persistent infection with oncogenic high-risk human papillomavirus (HPV) [1]. This malignancy usually has a gradual development through well-defined precursor stages, known as cervical intraepithelial neoplasia (CIN) grades 1, 2 and 3, before evolving into invasive carcinoma [4]. Most HPV infections are temporary in most people (transient), but if a high-risk HPV infection continues, it can cause a multi-step progression over years or decades from mild dysplasia (CIN I) to moderate and severe dysplasia (CIN II/III) and, finally, to invasive cancer [1]. This long precancerous phase provides opportunities for screening and intervention via Pap tests, HPV DNA testing, and treatment of CIN lesions before invasive carcinoma occurs.

The occurrence of oxidative stress in infected epithelial cells is a crucial component of HPV-driven carcinogenesis. By targeting p53 and retinoblastoma (RB) proteins, the viral oncoproteins E6 and E7 interfere with important tumor suppressor pathways, resulting in abnormal cell proliferation, a loss of cell cycle regulation, and a buildup of reactive oxygen species (ROS) [5]. The chronic oxidative stress that results speeds up the transition from precancerous CIN to invasive cancer by causing DNA damage, genomic instability, and mutations, promoting tumor growth [4]. Notably, HPV E6 effectively inactivates p53 by targeting it for ubiquitin-mediated degradation, which is why the TP53 gene is rarely altered in cervical malignancies, an exception among most cancers [4]. Wild-type p53 protects normal cells from oxidative damage by, for instance, triggering cell cycle arrest or death in response to DNA damage. However, HPV-infected cells lose this protection. Cervical carcinogenesis is fueled by the unregulated cell proliferation and oxidative stress caused by the combined impacts of HPV oncoproteins [6].

In diagnostic practice, several biomarkers have been implemented to improve the detection of high-risk cervical lesions. Transforming HPV lesions are characterized by enhanced proliferation indicated by Ki-67 and immunohistochemical overexpression of the cyclin-dependent kinase inhibitor p16^INK4a^, a surrogate marker of oncogenic HPV activity [7]. These markers are frequently employed together as a dual stain in pathology to differentiate benign formations from genuine high-grade CIN [7]. For instance, p16 and Ki-67 dual-staining cytology has demonstrated a high sensitivity in identifying CIN2+ lesions and aids in the triage of screening results that are positive for HPV [7]. These markers do have some specificity issues, though, and the higher sensitivity comes at the expense of more false positives, which may result in overtreatment or needless colposcopies. In other words, while p16 and Ki-67 greatly aid in identifying HPV-driven dysplasia, they cannot always reliably distinguish progressive lesions from regressive or transient HPV infections. These limitations highlight the need for novel biomarkers with better predictive accuracy to complement current screening and diagnostic algorithms.

In this regard, Reactive Oxygen Species Modulator 1 (ROMO1) is one new contender. A nuclear-encoded protein found in the inner mitochondrial membrane, ROMO1 is essential for controlling the generation of ROS and maintaining redox equilibrium [8]. ROMO1 can affect apoptosis, mitochondrial dynamics, and downstream oxidative signaling by adjusting the amount of ROS in the mitochondria. Abnormal ROMO1 expression has been linked in recent research to the emergence and spread of several solid cancers. ROMO1 is often overexpressed in malignancies such as lung, hepatocellular, colorectal, gastric, and glioblastoma. This overexpression has been linked to aggressive tumor characteristics such as increased invasion, metastasis, and worse patient survival [8]. Mechanistically, too much ROS produced by ROMO1 can stimulate redox-sensitive oncogenic pathways (NF-κB, MAPK/ERK, TGF-β), encourage the change of epithelium to mesenchymal tissue, and stimulate the migration and multiplication of cancer cells [8]. These results imply that ROMO1 may serve as a driver of carcinogenesis in addition to being a biomarker of tumor oxidative stress. To put it briefly, ROMO1 has become a possible marker of a high-ROS, high-risk tumor phenotype in a number of cancers.

Although oxidative stress, HPV, and cervical carcinogenesis have been linked, nothing is known about ROMO1’s involvement in cervical neoplasia. There is currently a lack of thorough information regarding the expression of ROMO1 in normal vs. precancerous lesions and cervical cancer, as well as on whether or not its expression is correlated with the severity of the disease. In order to define ROMO1 expression throughout the range of cervical squamous neoplastic development, the current study was created. We investigated ROMO1 protein levels using immunohistochemistry in invasive cervical squamous-cell carcinomas, cervical intraepithelial neoplasia (CIN I, II, and III) lesions, and healthy cervical epithelium. Our goal was to determine whether ROMO1 expression increases with higher-grade lesions and invasive disease, and to evaluate its clinical relevance as a potential biomarker of disease progression. Clarifying the pattern of ROMO1 expression in cervical neoplasia could provide new insights into HPV-related carcinogenic mechanisms and identify a novel biomarker to improve the specificity of cervical cancer diagnostics in the era of HPV-driven screening. Therefore, we investigated ROMO1 expression in normal cervical epithelium, CIN, and invasive carcinoma to determine whether oxidative stress markers correlate with disease progression.

## 2. Methods and Materials

This was a retrospective observational cohort study including all patients with histologically confirmed invasive cervical carcinoma diagnosed between 2015 and 2024 at the Department of Oncogynecology of Medical University Pleven. Ethical committee approval was obtained (number 656/29 June 2021) to investigate the role of ROMO1 in paraffin-embedded tumor tissue from those patients. Patients’ clinico-morphological information was retrieved from the Department’s electronic database. As this investigation included all eligible invasive cervical carcinoma cases diagnosed between 2015–2024, no a priori sample size calculation was performed.

### 2.1. Patient Characteristics

Characteristics of patients are presented in Table 1.

CIN lesions were categorized as LSIL (CIN I) or HSIL (CIN II/III). Invasive carcinomas were assessed for histologic subtype (SCC, AC, ASC), tumor grade (G1–G3), lymphovascular space invasion (LVSI), and nodal status (N0/N1), according to institutional pathology protocols.

**Definitions of key variables:**

- **LSIL/CIN I:** Dysplastic cells involving up to one-third of epithelial thickness.
- **HSIL/CIN II/III:** Dysplastic cells involving between one-third and two-thirds of the epithelium.
- **FIGO staging:** Classified according to the International Federation of Gynecology and Obstetrics (FIGO) 2018 cervical cancer staging system.
- **LVSI (lymphovascular space invasion):** Presence of tumor cells within endothelial-lined lymphatic or vascular channels.
- **N stage:** N0 = no regional lymph node metastasis; N1 = regional nodal involvement.

Patients were categorized based on their FIGO stage. Only individuals with lymph node metastases are seen in FIGO stage III (FIGO IIIC). Primary surgery was not advised for patients in stages FIGO IIIA and FIGO IIIB of the CC therapy protocol at this time.

### 2.2. Immunohistochemical Methods

We performed immunohistochemistry on one 3 µm thick slide for each patient studied, having previously evaluated the slide with hematoxylin eosin staining. We used an ROMO1 antibody-clone OTI2C12, Mo, dilution 1:150 (Abcam, Cambridge, UK), and visualization with an EnVision™ FLEX, High pH system using AutostainerLink 48 (Dako, Glostrup, Denmark). According to the manufacturer’s protocol, we performed heat-mediated antigen retrieval with citrate buffer pH 6 before starting the IHC staining.

#### Immunohistochemical Scores

There are still no accepted methods for reporting ROMO1. We used H-score immunohistochemical methods to analyze antibody expression in cases with invasive carcinoma, as described in detail in our previous article [9]. When determining positivity, only cytoplasmic staining was included.

We assessed the expression of ROMO1 in biopsy samples from normal cervix (without cellular atypia in the epithelium) and from those with dysplastic epithelium (including low-grade squamous intraepithelial lesion—LSIL/CIN I and high-grade squamous intraepithelial lesion—HSIL/CIN II/CIN III). We evaluated the intensity and proportion of positivity throughout the thickness of the epithelium. In these cases, we can assess the degree of intensity (weak, moderate, strong) in tissues without atypia and with CIN. However, unlike in invasive carcinomas, we cannot make a semi-quantitative assessment of ROMO1 expression, because in normal tissue the preinvasive diagnosis by itself does not include the ability to determine tumor area, and does not allow the percentage of positive cells to be determined. For this reason, we determined the part with positive cell layers relative to the entire thickness of the covering epithelium.

**Figure 1:**
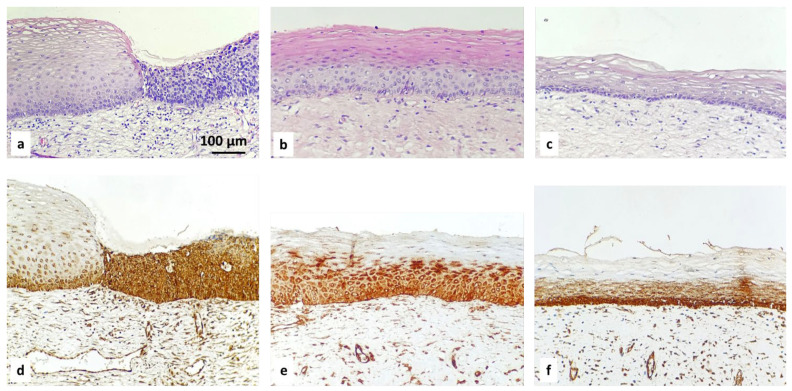
Hematoxylin and eosin staining (**a**–**c**) and IHC staining with ROMO1 (**d**–**f**): normal squamous epithelium (on the left of the picture (**a**,**d**)); HSIL—CIN III (on the right of the picture (**a**,**d**)); HSIL—CIN II (**b**,**e**); LSIL—CIN I (**c**,**f**); magnification ×200.

**Figure 2:**
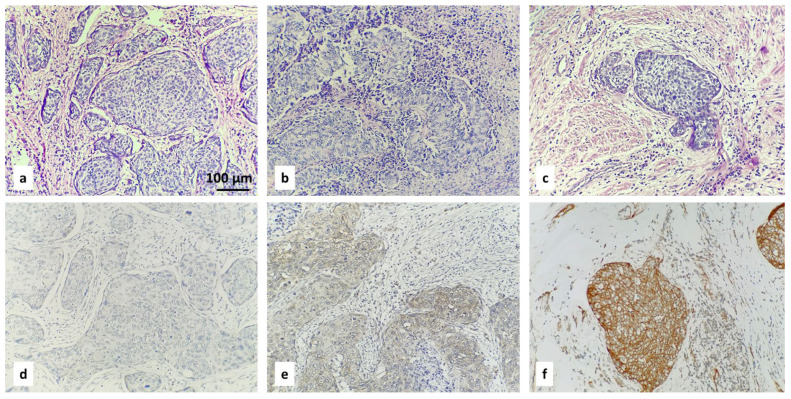
IHC staining with ROMO1 in cervical squamous-cell carcinoma: negative result (**d**), low (**e**) and high (**f**) H-scores. Corresponding hematoxylin- and eosin-stained tissue samples are above (**a**–**c**); magnification ×200.

**Figure 3:**
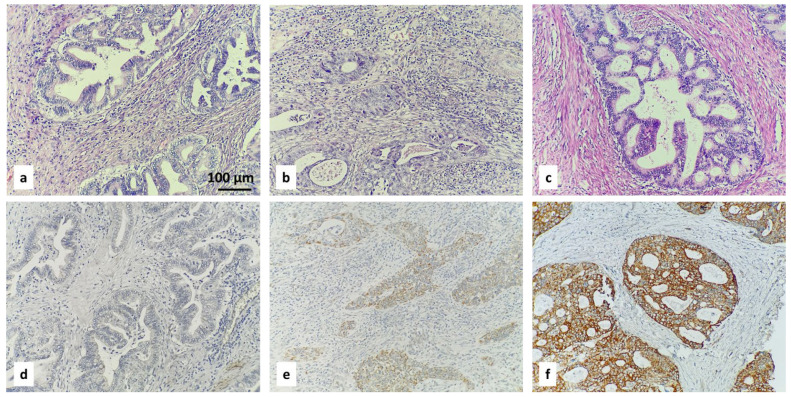
IHC staining with ROMO1 in cervical adenocarcinoma: negative result (**d**), low (**e**) and high (**f**) H-scores. Corresponding hematoxylin- and eosin-stained tissue samples are above (**a**–**c**); magnification ×200.

**Figure 4:**
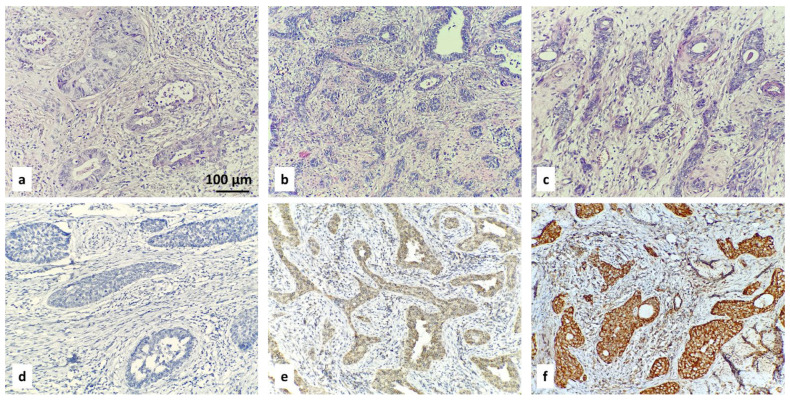
IHC staining with ROMO1 in cervical adenosquamous carcinoma: negative result (**d**), low (**e**) and high (**f**) H-scores. Corresponding hematoxylin- and eosin-stained tissue samples are above (**a**–**c**); magnification ×200.

Examples of the IHC-specified categories are illustrated in Figure 1, Figure 2, Figure 3 and Figure 4.

##### H-Score

For H-score assessment, the following formula was applied:

ROMO1 H-score = (% of cells stained at weak intensity × 1) + (% of cells stained at moderate intensity × 2) + (% of cells stained at strong intensity × 3). The resulting scores ranged from 0 to 300, where 300 was equal to 100% of tumor cells stained strongly (3+). Expression level was categorized according to the median value of the H-score: low (with H-score ≤ 68.40) or high (with H-score > 68.40). When <1% of positive cells had H-score = 0, this was considered to be a negative result. The three categories of negative result, low H-score, and high H-score are included in Figure 2, Figure 3 and Figure 4.

### 2.3. Statistical Analysis

ROMO1 H-scores were-tabulated against key clinical and pathological parameters: tumor (T) stage, nodal (N) stage, histological subtype, and lymphovascular invasion (LVSI). The resulting contingency tables were analyzed using a chi-squared test. Comparison of age distribution per ROMO1 H-scores was achieved using two-sided *t*-tests. *p*-values of <0.05 were considered statistically significant. All statistical analyses were performed using R version 4.5.0 (2024, R Foundation for Statistical Computing, Vienna, Austria).

Plots were generated using the ggpubr (version 0.6.1) and ggplot2 (version 3.5.2) packages for R.

## 3. Results

### 3.1. Immunohistochemical Analysis

I. ROMO1 in normal tissue from cervix
  - Positive basal expression with strong intensity in normal cervical epithelium.
II. ROMO1 in squamous intraepithelial lesions (SIL): LSIL, HSIL
  - Diffuse suprabasal expression with moderate to strong intensity in the area of abnormal cells in all CIN cases:
    - LSIL/CIN 1: Refers to abnormal cells affecting about one-third of the thickness of the epithelium;
    - HSIL/CIN 2: Refers to abnormal cells affecting about one-third to two-thirds of the epithelium;
    - HSIL/CIN 3: Refers to abnormal cells affecting more than two-thirds of the epithelium.

### 3.2. ROMO1 Expression and Clinicopathologic Features in Invasive Carcinoma

**Figure 5:**
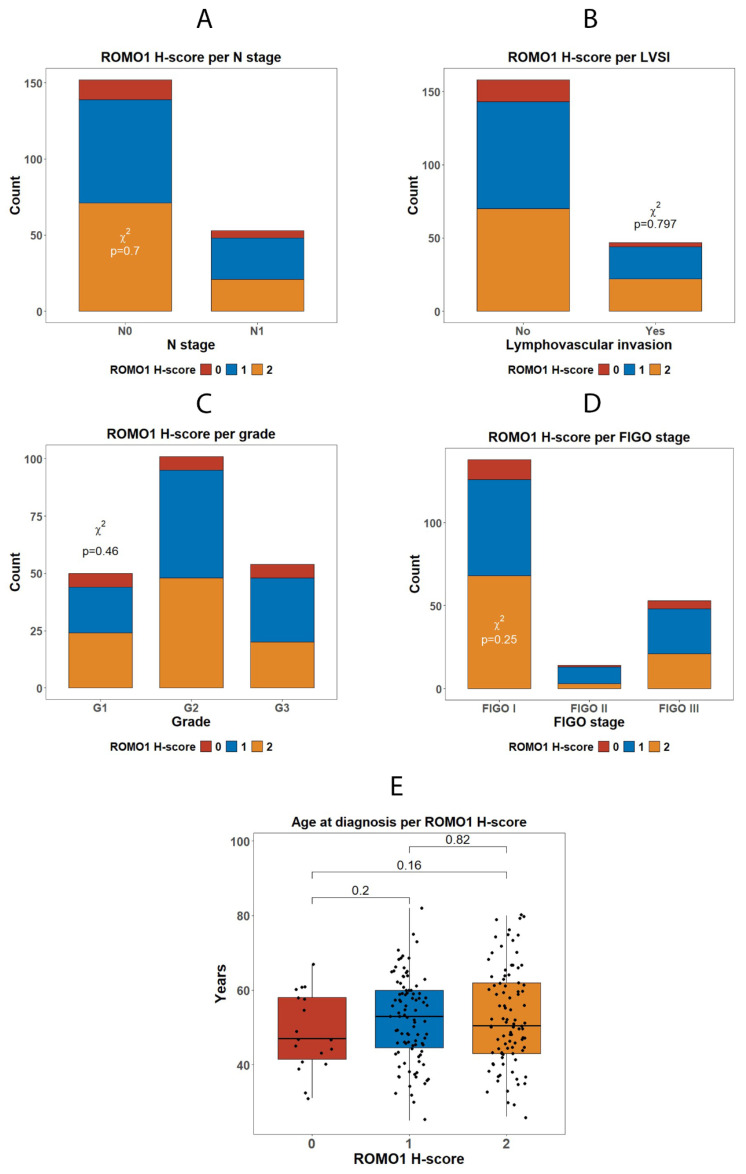
Association of ROMO1 expression with clinicopathological variables and patient age in invasive cervical carcinoma. (**A**–**D**) Distribution of ROMO1 H-scores (0 = negative, 1 = weak, 2 = strong) across clinicopathological parameters. No statistically significant associations were observed between ROMO1 expression and (**A**) nodal status (N stage; *p* = 0.70), (**B**) lymphovascular space invasion (LVSI; *p* = 0.797), (**C**) tumor differentiation grade (*p* = 0.46), or (**D**) FIGO stage (*p* = 0.25) using χ^2^ tests. ROMO1 overexpression was prevalent across all subgroups, suggesting that oxidative imbalance is a general feature of cervical carcinoma independent of traditional prognostic factors. (**E**) Relationship between patient age and ROMO1 H-score at diagnosis. Median age did not differ significantly among groups with low, intermediate, or high ROMO1 expression, indicating that ROMO1 levels are unrelated to age at presentation.

**Figure 6:**
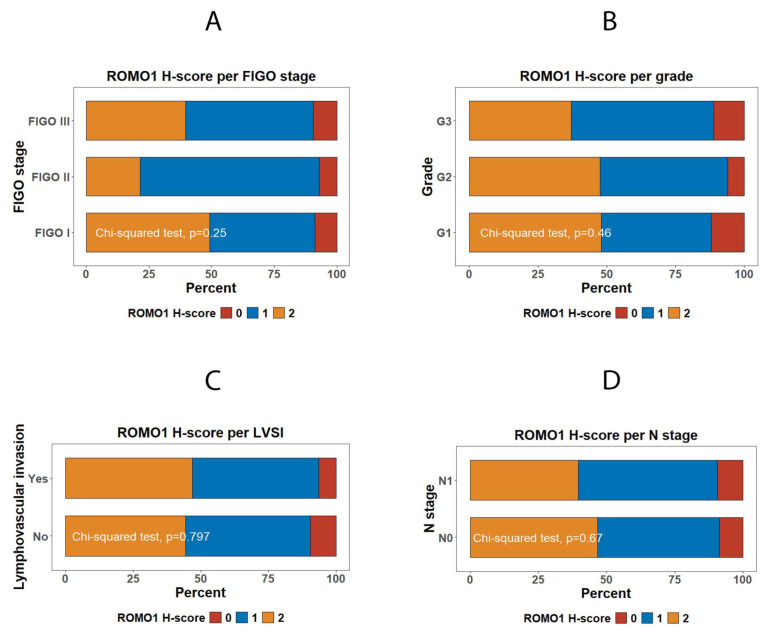
ROMO1 expression according to clinicopathological parameters in invasive cervical carcinoma. (**A**–**D**) Proportional distribution of ROMO1 H-scores (0 = negative, 1 = weak, 2 = strong) across clinicopathological variables: (**A**) FIGO stage, (**B**) tumor grade, (**C**) lymphovascular space invasion (LVSI), and (**D**) nodal status (N stage). No statistically significant associations were found between ROMO1 expression and any of these parameters (χ^2^ tests, *p* = 0.25, *p* = 0.46, *p* = 0.797, and *p* = 0.67 for FIGO stage, tumor grade, LVSI, and N stage, respectively).

In our cancer cohort (*n* = 205), ROMO1 H-score showed no significant associations with the following (Figure 5 and Figure 6):

(1) FIGO stage I vs. II vs. III (χ^2^ *p* = 0.25)
(2) Histologic grade G1 vs. G2 vs. G3 (χ^2^ *p* = 0.46)
(3) Lymphovascular invasion (no vs. yes; χ^2^ *p* = 0.80)
(4) Nodal status N0 vs. N1 (χ^2^ *p* = 0.67)
(5) Patient age (≤50 y vs. >50 y; χ^2^ *p* = 0.38)

**Figure 7:**
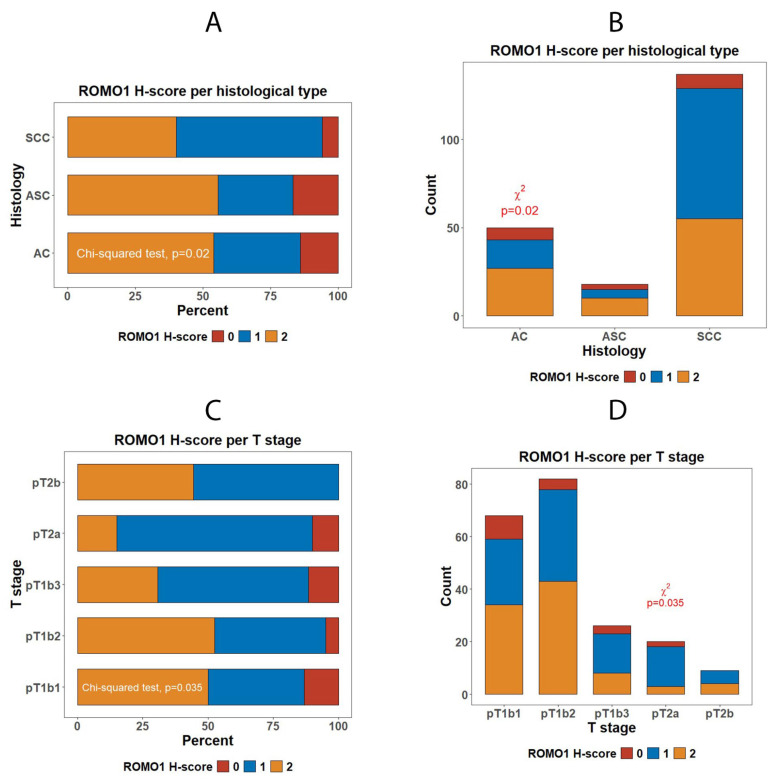
Distribution of ROMO1 expression by histological subtype and primary tumor stage in invasive cervical carcinoma. (**A**,**B**) ROMO1 H-score distribution across histological types. Squamous cell carcinomas (SCCs) show significantly higher ROMO1 expression compared with adenocarcinomas (ACs) and adenosquamous carcinomas (ASCs) (χ^2^ = *p* = 0.02). (**C**,**D**) ROMO1 H-score distribution by pathological tumor (pT) stage. A significant decline in high-ROMO1 cases is observed with increasing tumor size and stage (χ^2^ = *p* = 0.035), demonstrating an inverse correlation between ROMO1 expression and tumor progression.

ROMO1 expression did vary as follows:

(1) ROMO1 expression differed significantly between histologic subtypes, with SCC showing higher expression than AC and ASC (*p* = 0.02). High ROMO1 immunoreactivity was most frequent in SCC, followed by ASC, and lowest in AC.
(2) With regard to depth of invasion (pT stage) (pT1b1–pT2b; χ^2^ *p* = 0.035), we concluded that ROMO1 expression varied significantly across pT subcategories (pT1b1, pT1b2, pT1b3, pT2a, pT2b; χ^2^ *p* = 0.035). High H-score [2] was most frequent in pT1b2 (52%) and least in pT2a (11%), with intermediate levels in other groups (Figure 7).

## 4. Discussion

This study provides a broad overview of how ROMO1 behaves across the main stages of cervical disease. By examining normal epithelium, CIN lesions, and invasive tumors together, we were able to observe a clear shift in expression that follows the progression of HPV-related pathology. ROMO1 was essentially absent in normal squamous epithelium, aside from minimal reactivity in the basal layer, increased steadily through CIN I–III, and then showed lower and more variable levels once invasive cancer had developed. This trajectory suggests that ROMO1 is most active during the early stages of neoplastic change, when oxidative imbalance becomes a more prominent feature of HPV-driven transformation.

### 4.1. ROMO1 in CIN

The uniformly strong expression of ROMO1 in CIN II and CIN III underlines the importance of oxidative stress at this stage of disease. CIN represents a period of active viral interference with epithelial homeostasis, during which HPV oncoproteins alter cell cycle regulation and increase the production of reactive oxygen species [10]. Our findings do not explore the mechanistic basis of this relationship, but the progressive increase in ROMO1 across CIN grades supports the concept that mitochondrial redox activity rises as lesions become more atypical.

### 4.2. ROMO1 in Invasive Cancer

In contrast to CIN, invasive tumors did not show a continued rise in ROMO1 expression. Instead, ROMO1 staining was strongest in early-stage tumors and became less common in larger or more deeply invasive lesions. This pattern differs from what has been reported in several non-HPV-related cancers, where high ROMO1 expression is usually associated with more advanced disease [11]. Because we did not investigate molecular pathways in this study, any explanation would be speculative, and we have avoided making mechanistic claims. Our findings simply show that ROMO1 does not track with disease severity in cervical cancer in the way it does in HPV-independent tumors.

### 4.3. Subtype Differences

ROMO1 expression also varied across histologic subtypes. Squamous carcinomas showed higher levels on average than adenocarcinomas or adenosquamous tumors. Whether this reflects differences in HPV genotype distribution, the biology of keratinizing epithelium, or other microenvironmental factors cannot be determined from the present data. The observation is noteworthy, however, and suggests that ROMO1 may not behave uniformly across all cervical cancer types.

### 4.4. Diagnostic Considerations

The marked difference between normal epithelium and CIN, together with the graded increase across CIN categories, indicates that ROMO1 may help distinguish reactive epithelial changes from true dysplasia. It could potentially complement markers already used in clinical practice, such as p16 and Ki-67, particularly in cases where morphology is ambiguous. Further work is needed to determine how ROMO1 might be incorporated into diagnostic algorithms.

When evaluating patients with postmenopausal bleeding, endometrial cancer must always be ruled out first. Transvaginal ultrasound remains the primary imaging method in this setting before any cervical pathology is considered [12]. This broader diagnostic context is important when discussing any biomarker proposed for cervical disease.

### 4.5. Study Limitations

Several limitations must be acknowledged. Immunohistochemistry provides semi-quantitative information and is subject to some observer variability, although consistent scoring criteria were used. The study reflects the experience of a single institution and may not represent the full diversity of cervical cancer cases. Most importantly, we did not have sufficient follow-up to evaluate recurrence or survival outcomes. As a result, we cannot draw conclusions regarding the prognostic value of ROMO1, and future longitudinal studies will be required to address this question.

## 5. Conclusions

Taken together, our data identify ROMO1 as a dynamic biomarker of cervical neoplasia, marking the oxidative surge of early transformation and its attenuation with tumor advancement. Unlike in other cancers where ROMO1 tracks aggressiveness, in cervical cancer its decline reflects viral reprogramming and metabolic adaptation. These insights refine our understanding of HPV-driven redox biology and open new avenues for biomarker development and redox-targeted prevention strategies in cervical disease.

## Figures and Tables

**Table 1:** Characteristics of patients.

**Healthy cervix**	30	100
**Precancerous lesion**	41	100
LSIL	6	14.6
HSIL	35	85.4
**Age**	205	100
>50	106	51.7
≤50	99	48.3
**T stage**		
T1b1	68	33.17
T1b2	82	40
T1b3	26	12.6
T2A	20	9.75
T2B	9	4.39
**N stage**		
N0	152	74.14
N1	53	25.85
**FIGO stage**		
FIGO I	138	67.3
FIGO II	14	6.82
FIGO III	53	25.85
**Histology**		
AC	50	24.4
ASC	18	8.78
SCC	137	66.8
**Grade**		
G1	50	24.39
G2	101	49.26
G3	54	26.34
**LVSI**		
Yes	47	22.92
No	158	77.1
**Total**	205	100

Expression of ROMO1 with moderate to strong intensity in all cases of CIN. LSIL/CIN 1 refers to abnormal cells affecting about one-third of the thickness of the epithelium. HSIL/CIN 2 refers to abnormal cells affecting between one-third and two-thirds of the epithelium. HSIL/CIN 3 refers to abnormal cells affecting more than two-thirds of the epithelium.

## Data Availability

The original contributions presented in this study are included in the article. Further inquiries can be directed to the corresponding author.
